# Factors influencing early withdrawal from a drug and alcohol treatment program and client perceptions of successful recovery and employment: a qualitative study

**DOI:** 10.1186/s12888-018-1864-y

**Published:** 2018-09-18

**Authors:** Tarran Prangley, Sabrina Winona Pit, Trent Rees, Jessica Nealon

**Affiliations:** 1 School of Medicine, University of Wollongong, Wollongong, Australia; 2 School of Medicine, University Centre for Rural Health, Western Sydney University, 61 Uralba Street, Lismore, NSW 2480 Australia; 3 Sydney Medical School, University of Sydney, 61 Uralba Street, Lismore, NSW 2480 Australia; 4 The Buttery, Bangalow, Australia; 5 Faculty of Science, Medicine and Health, School of Medicine, University of Wollongong, Wollongong, Australia

**Keywords:** Therapeutic community, Substance use disorders, Addiction, Early exit, Recovery, Employment

## Abstract

**Background:**

Substance use disorders are a major contributor to the economic and healthcare burden in Australia. Therapeutic communities (TCs) are utilised treatment methods globally, though low program completion rates continue to represent a major obstacle in effective and sustainable drug and alcohol treatment. The aim of this study was to explore reasons for early withdrawal from TC programs and perceptions of successful recovery. This study also aimed to explore how employment and volunteering related to early exit and perceptions of successful recovery.

**Methods:**

Semi-structured qualitative interviews were conducted with 13 ex-residents from a long-term TC program at a community-based rehab organisation in regional Australia.

**Results:**

Thematic analysis revealed a complex interplay of factors contributing to early TC withdrawal, and perceptions of successful recovery from a lived experience perspective and how this was shaped by employment and volunteering. Eleven themes were identified. Three relating to reasons for joining the program, which connected with ultimate withdrawal from the program: Pre-program existing relationships, pre-program employment situation and needing a ‘circuit breaker’ in their life. Three relating to reasons for early withdrawal: TC program characteristics, relationships during the program and planning future employment. Five relating to perceptions of successful recovery: Improved understanding of their addiction, reduced substance use, improved physical and psychological health, relationship success and employment success.

**Conclusions:**

Reasons for leaving treatment early are multi-faceted and revolve around relationships, planning future employment and program characteristics. The influence that each plays on their decision to leave early is varied and determined by the value they assign it. Perceived success extends far beyond achieving and maintaining abstinence to encompass improved relationships, psychological and physical wellbeing, understanding of addiction and employment, studying or volunteering. Self-worth and feeling able to contribute to society through employment, study and volunteering were perceived to be essential elements of successful recovery. Clinicians, policy makers and program developers should use the extended definition of successful recovery from the ex-clients perspective when determining the clinical and economic effectiveness of TC programs.

## Background

Substance use disorders are a major contributor to the economic and healthcare burden in Australia; costing $55 billion annually through crime, road accidents, workplace productivity losses and healthcare costs [1]. The National Drug Strategy [2] identified alcohol, cannabis and methamphetamine use as priority areas*.* Improvements in Drug and Alcohol (DA) treatment services help to address these issues, particularly by targeting demand reduction, peer-education networks, social competence training and increasing community engagement.

Therapeutic communities (TCs) are drug-free residential rehabilitation programs where clients live together in a hierarchal-based supportive environment with increasing levels of social and personal responsibility [3]. This treatment model is utilised in DA rehabilitation with the aim to socially rehabilitate drug users through personal change and skills training [4].

Success of these programs has been evaluated through a literature review of 16 controlled TC studies [5]. TC residents had superior employment outcomes and improved relationships and psychological functioning compared to controls. Length of stay in treatment and participation in subsequent aftercare were found to be consistent predictors of recovery. Contrary to these findings, a systematic review of residential treatment studies did not show superior employment outcomes in TC residents [4]. However, only one of the 11 studies included in the latter study measured employment as an outcome variable, and this study did find higher employment in the TC group at follow-up [4]. Improvements in other outcome measures included substance use, criminal activity, mental health and social engagement.

Low program completion rates, ranging from 9 to 56%, continue to represent a major obstacle in effective and sustainable DA treatment [3, 6]. Length of stay in treatment is associated with higher levels of abstinence, reduced crime and unemployment, and improved quality of life [3, 7]. Predictors of early withdrawal have therefore been explored to identify clients who may be at increased risk of dropout and enable more appropriate referral to TC services for clients who are most likely to benefit from the program.

Three quantitative studies investigated predictors of TC completion [8, 9]. Disadvantageous decision making, lower perception of likeliness to complete the program, recent prison release, aggression, self-harm or suicidality, relationships, amphetamine use and younger and older age, were all found to be predictors of early withdrawal. However, some discrepancies exist in the literature. Harley et al. found amphetamine use to be a negative predictor of program completion (Harley M, Pit SW, Rees T, Thomas S: Completion rates and psychosocial intervention effectiveness in an Australian substance abuse therapeutic community, submitted.), contrary to Darke et al. [9] who found no association with primary drug use. The relationship between disadvantageous decision making and residential treatment drop-out investigated by Stevens et al. [8] may have been mediated by potential confounders not investigated, including psychiatric comorbidities and motivation for treatment. Additionally, although the study concerned a residential treatment population, the treatment facilities did not apply the ‘community as a method’ approach typical for TCs. Issues with generalisability beyond cocaine dependent individuals also limits Stevens et al.’s [8] research.

A qualitative approach enables exploration of the complex relationships between client variables, treatment retention, and re-integration into society from the clients’ perspective. Despite the benefit, there is a deficit of qualitative research exploring reasons for early withdrawal from adult TCs. Two qualitative studies investigating adolescent TC populations identified negative relationships with staff as major contributors to early withdrawal [10, 11]. Landrum et al. [10] investigated reasons for early withdrawal through five focus groups among judicially referred adolescents, parents and treatment staff. Five main domains affected retention: relationships, responsibility, emotional regulation, thinking and self-efficacy. The importance of motivation for treatment success was also noted.

Chen et al. [11] used semi-structured interviews to investigate the experiences of 11 adolescents who dropped out of an Orthodox Jewish TC, focusing on reasons for early TC withdrawal and recovery. Chen et al. [11] found antagonistic staff and adolescent interactions, lack of motivation and program dissatisfaction, and a desire to make positive changes in various aspects of their life. However, like Landrum et al. [10] the narrow focus on Orthodox Jewish adolescents and judicially referred adolescents limits its generalisability to other populations.

Most TC research focuses on abstinence as a surrogate for program success, however little research has investigated what recovery success looks like for clients. Aslan [12] aimed to address the question ‘when is “unsuccessful” successful?’ through semi-structured interviews with 13 service users who withdrew early from three TCs in the United Kingdom. Recovery success was found to manifest in various ways including improvements in psychological well-being, relationships, accommodation and employment, as well as reductions in relapse and reoffending. Eighty-five percent of participants reported using their time in positive ways such as voluntary and paid employment, college and spending time with friends or at support services. Most participants were also actively involved in further treatment after discharge.

Aslan [12] highlights an important question about redefining recovery ‘success’ to better reflect the clients’ perspective. Similarly, Laudet [13] investigated what recovery success looks like from a lived-experience perspective through interviews with 354 individuals with resolved substance use disorders, not specifically individuals who left residential rehabilitation early. Abstinence was identified as necessary for recovery, though recovery goals extended well beyond abstinence. Recovery was seen by most as a process of growth through which the individual achieved improved financial and living conditions, social networks and physical and/or mental health.

There is a considerable lack of qualitative research, and, to our knowledge, none in an Australian context, exploring both reasons for early exit and successful recovery from a lived-experience perspective. The current study aimed to gain a better understanding of the reasons for early withdrawal from TC programs and perceptions of successful recovery. A secondary aim was to explore how employment and volunteering related to early exit and perceptions of successful recovery.

## Method

### Participants

Participants comprised ex-residents from a long-term TC program at a community-based rehab organisation located in regional Australia. All participants left the TC program early, either voluntarily or involuntarily, and had given consent to be contacted for future research.

### Recruitment

Participants were informed about the project via email from the organisation, and given the opportunity to advise if they did not wish to be contacted by the researchers. Recruitment was targeted to ensure demographic diversity. In particular, a varied sample of age groups, gender and voluntary or involuntary withdrawal from the program was sought.

### Data collection

Participants were contacted by author TP via telephone or email and provided an information sheet with the goals of the research. No prior relationship was established prior to study commencement between the participants and TP. Prior to the interview, participants were asked to sign a consent form or give verbal consent over the telephone. Participants were also provided with resources to contact should any distress arise from the interview.

The semi-structured interview schedule was developed from the literature and in consultation with the staff. Questions were designed to explore factors related to reasons for early withdrawal, substance use and treatment history, employment, social supports and perceptions of recovery. The interview schedule was pre-tested for clarity and appropriate question ordering, and further refined where necessary.

The interviews were conducted by TP via telephone from October 2017 to December 2017. TP has a bachelor degree in psychology and has received training in interviewing techniques as part of a psychology and medical degree. Author TR provided content expertise through explaining the context of the programs. Further guidance in qualitative data analyses was provided by author SWP. Telephone interviews were conducted as the majority of participants now lived inter-state, making face-to-face interviews unfeasible. Data saturation was achieved and no new themes emerged in the final two interviews.

#### Analysis

Reflective notes were kept during the interview process to guide analyses. Interviews were audio-recorded, transcribed verbatim and analysed thematically [14]. Microsoft® Word was used throughout the data analyses process. The first transcript was read and analysed by TP and SWP and preliminary codes were generated after discussion. The code book was independently trialled by TP and SWP on two interviews, followed by an in-depth discussion to reach a consensus on coding. The coding tree was broadly defined into life prior to joining the drug rehabilitation program, time during the program, perceived success and factors influencing perceived success. TP coded the remaining transcripts. Coded data extracts were then collated together and emerging themes identified. Themes were discussed initially between TP and SWP, and then refined to ensure they captured the meaning of the data.. The themes were then fed back to TR to further aid in data interpretation and potential theme refinement. Data saturation was discussed between TP and SP and it was felt that data saturation was achieved after eleven interviews but two more interviews were conducted to confirm. Transcripts were not returned to participants for comment and/or correction. Quotes have been used in discussion of each topic.

#### Ethics

Ethics approval for this project was granted by Western Sydney University Human Research Ethics Committee (HREC No: H11353) and the University of Wollongong Human Research Ethics Committee (Ethics Number 2017/404).

## Results

Thirty-five residents who left the TC program early were contacted. Reasons for non-participation were: inability to contact due to a disconnected or incorrect telephone number or email address (*n* = 7), no reply to telephone or email contact (*n* = 13), not wishing to partake (n = 1) and unable to be contacted after initial positive contact (n = 1). Thirteen interviews were conducted (7 male and 6 female). The age range was 31–61 years, mean age 44 years. The interviews varied in length, ranging from 15 to 39 min (average 27 min). Participant demographics are listed in Table 1.

**Table 1:** Demographics of participants

		Males	Females	Total number of participants (%)
Participants		7	6	13 (100%)
Age	31–34	0	3	3 (23%)
	35–39	0	2	2 (15%)
	40–44	2	0	2 (15%)
	45–49	2	0	2 (15%)
	50–54	1	1	2 (15%)
	55–61	2	0	2 (15%)
Reason for early exit from TC program	Voluntary	3	5	8 (62%)
	Involuntary^a^	4	1	5 (38%)
Primary dependence prior to admission^b^	Alcohol	6	0	6 (46%)
	Drug	1	3	4 (31%)
	Polysubstance	0	3	3 (23%)
Length of stay	< 2 weeks	1	0	1 (8%)
	2–4 weeks	0	2	2 (15%)
	5–8 weeks	1	3	4 (31%)
	9–12 weeks	1	1	2 (15%)
	13–16 weeks	0	0	0 (0%)
	> 16 weeks	1	3	4 (31%)

^a^The reasons for involuntary withdrawal included breaking the rules, such as bringing or purchasing illicit substances, contacting family outside of the program, having a relationship with another resident, and arguments with staff

^b^Substances included heroin, ice, cocaine, cannabis and alcohol. Prescription medication use included benzodiazepines and codeine

**Fig. 1:**
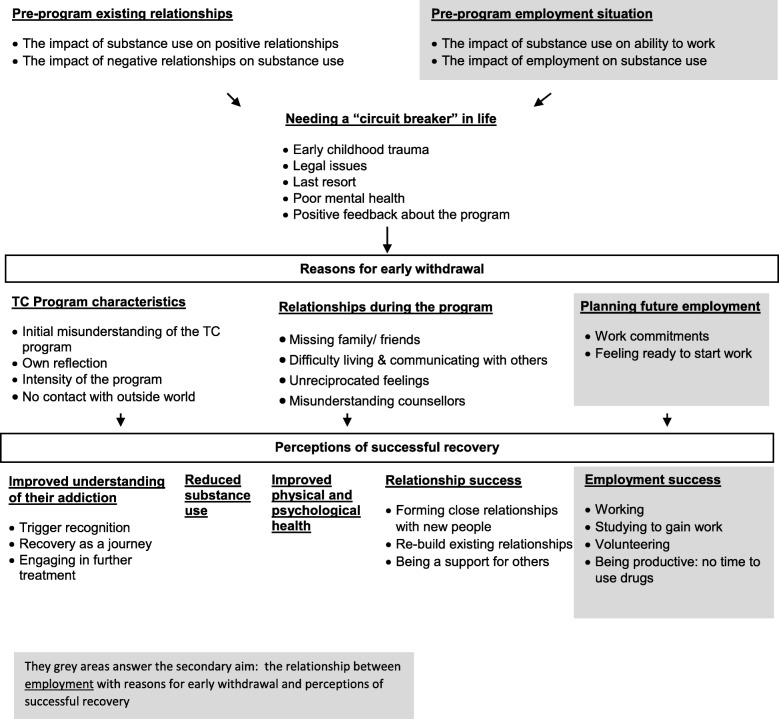
Themes

The main themes and their complex relationships are depicted in Fig. 1. Figure 1 demonstrates that pre-existing relationships and participant’s employment situation prior to program entry were linked with participant’s need for a ‘circuit breaker’ in their life. The themes needing a ‘circuit breaker’ in life, pre-existing relationships and pre-program employment situation reflected the reasons for entry into the program, which was connected with ultimate withdrawal from the program. Furthermore, Fig. 1 shows that reasons for early withdrawal centred around three major themes: TC program characteristics, relationships during the program and planning for future employment. Finally, five themes emerged that participants perceived to be part of a successful recovery: improved understanding of their addiction, reduced substance use, improved physical and psychological health, positive relationships with others and employment success. Both relationships and employment were themes that played a major part before, during and after entering the TC program and influenced participant’s reasons for withdrawing early and their perceptions of successful recovery.

### Reasons for joining the program

Both existing relationships, and pre-program employment situations were linked with the need for a circuit breaker in life and formed the basis for participants’ perceived need to enter the TC program.

#### Pre-program existing relationships

Support networks prior to the TC program were varied. Whilst some reported positive relationships with family and friends, they also felt that their supports did not understand their journey. Others described a strong sense of isolation in the later stages of their addiction and whilst some may have had support around them at this time, it was not recognised.

‘…when I was in the end of my addiction I didn’t really speak to anyone…my family begrudgingly communicated with me. But I didn’t really have proper conversations with anyone.’

For others, craving an emotional connection of any form resulted in maintaining destructive relationships, such as other substance users, which negatively influenced their addiction.

‘……<family member> and I used a lot of ecstasy… smoked heroin once together. This is just completely what you don’t do with your <family member>, ever.’

#### Pre-program employment situation

The influence of employment on substance abuse is complex. For most, periods of employment were interspersed with long periods of unemployment and lack of stability. For one participant, her work played such an integral part in her identity that its’ loss directly influenced her to relapse. Others reported poor decision-making and lacked the skills to maintain jobs.

‘…I was really good at getting jobs and really shithouse at keeping them.’

For twelve participants, employment allowed access and means to maintain their addiction, such as working in a drug and alcohol environment like clubs and pubs, or working in high paying jobs like law and finance. For others, working alone or in remote and rural locations with no oversight allowed for their addiction to continue unnoticed at work.

‘I ran nightclubs for a long time … I was given a lot of free cocaine. Um and a lot of free everything…’

### Needing a ‘circuit breaker’ in life

Participants talked about several factors leading to them joining the program. The reasons for joining the program set the scene for being able to talk about their life in relation to the program itself, their reasons for leaving and their subsequent perceptions of successful recovery. Reasons for joining the program were mainly based on negative experiences such as early childhood trauma, being in trouble with the law, feeling that attending the program was their last chance in life and poor mental health. A positive factor was that some had heard positive feedback about the program. Eleven participants had a history of life-long challenges. Most participants described early life challenges such as childhood trauma and substance use, isolation and complex mental health issues. For most, their substance use began in middle childhood or early adolescence as a means of escaping reality.

‘… anything you’d put in front of me that would change the way I felt and make me feel a bit better I’d take it.’

Further challenges included drug-related offences and legal issues, with three participants having spent time in prison.

‘I went to prison when I was 23 as a result of my drug using behaviours.’

Twelve participants described a turning point that led to their decision to apply for the program. Some described feeling desperate and fearful of the consequences of continued addiction and feeling that their life was on the line. Others felt isolated and crippled by poor mental health.

‘If I hadn’t had gone through the <rehab organisation> I’d probably be dead to be honest.’

For others, decisions to attend the rehab organisation were influenced by hearing positive things about the programs from previous residents, or being referred by other treatment facilities because of their long addiction history.

### Reasons for early withdrawal

Reasons for withdrawal revealed three major themes: the TC program characteristics, relationships and planning for future employment. Firstly, TC characteristics that led to participants leaving early varied, including a misunderstanding of the program and different expectations of the program when they joined. Some had time during the program to reflect on their life and substance use and mentioned they were able to come to the conclusion that the program was not for them or they felt they had learnt enough during the program and were ready to leave early. Some participants did not cope with the intensity of the program and relationships with others within the program such as being with others 24/7. On the contrary, other participants reported feeling very isolated from the outside world such as friends and family and therefore left the program early. The intensity of the program and the rule of no contact with the outside world during the program has a clear link with participants’ relationships with others and their reasons for leaving early.

#### TC program characteristics

Four participants reported an initial misunderstanding about the TC program rules or duration. This misunderstanding had positive consequences for some whom otherwise may not have attended the program had they fully comprehended the rules.

‘I was very naïve um about what I was getting myself in for, but that was good in a way.’

For others, a better understanding of the program duration may have allowed arrangements to be made to stay longer.

Some participants found the intensity of the TC program to be very challenging and confronting. Some described the therapy as “tiring and relentless”, and found it difficult to be open about their feelings and deal with past traumas.

‘… the program’s very tiring… necessary but it’s work… it’s therapy all day, all night it’s relentless.… it’s 24/7.’

Others felt uncomfortable openly confronting other residents about disagreements. One participant described a ‘shame and blame’ environment. Another attributed their decision to leave the program early to the ‘informant culture’. Some disagreed with the program rules, and one participant described the program as ‘cultish’. However, on reflection of their experiences, most developed an appreciation for the rules and the commitment of those who stayed.

‘There were rules that I found really odd. Um but now I understand how the program works.’

For others, the decision to leave early was self-guided. Some felt they were adequately equipped and were ready to leave. Others had just had enough and expressed feelings of guilt for leaving the program early.

All participants discussed feeling isolated from the outside world. Whilst this isolation was challenging, it was also recognised as necessary. For some, isolation provided a safe environment away from violent relationships and temptations.

‘ … I needed to be taken totally out of my environment … out of the people, places and things that I was associated with, and dropped into a therapeutic community where I could recover…’

For others, isolation from their support network outside of the TC program constituted a major reason for leaving early.

#### Relationships during the program

For some, missing family and friends influenced their decision to leave early. Relationships formed with staff and residents within the program were challenging. In particular, some reported difficulties living and communicating with other people. Two participants developed feelings for others that were not reciprocated and one participant was asked to leave after breaching a program rule; starting a relationship with another resident. Some felt that the counsellors could have been more understanding and others felt like the counsellors weren’t supportive. However, despite these difficulties, on reflection most participants recognised the necessity of the isolation and relationships within the program.

**‘…**everyone’s their brother’s keeper and everyone’s brother’s policeman.’

Conversely, for others the support of family and friends served as a motivating factor to stay in the program, as did the support of program staff and residents. Several reported the therapeutic value of staff also being recovering addicts and seeing them as a positive role model. Living and interacting with people who had faced similar challenges to them helped to foster a sense of belonging within the community, and some reported feeling accountable to the therapeutic community.

‘… I just felt for the first time that I had love around … and I had a massive amount of support.’

#### Planning future employment

Work commitments outside the program also influenced the decision to leave the program early. For some this was having a job to go back to and for others this was feeling ready to find a job and start working.

‘…my employer in <city> had given me time off.’

### Perceptions of successful recovery

Participants discussed their life after leaving the TC program and several themes emerged that demonstrated a variety of important elements of successful recovery which will be discussed below.

#### Improved understanding of their addiction

Ten participants reported gaining a better understanding of their addiction triggers and barriers to ongoing recovery.

‘… I didn’t really want to give up that <living-place> but I understand that it was part of my recovery you know because it was a trigger with everyone using in that house …’

Six participants learnt that ‘recovery is a journey’. For some this involved sharing their own and other people’s stories, whereas others felt isolated in their journey.

As part of ‘recovery as a journey’, most participants recognised the importance of ongoing treatment. All participants had engaged in further drug and alcohol treatments since leaving the program. The treatment varied from NA and AA support groups, counselling and doctors, medications and alternative therapies such as yoga and meditation. Most participants had engaged in a multi-faceted treatment approach.

‘I’ve done a little bit of work with a psychologist. I’ve worked the steps. I’ve done you know I’ve explored meditation, acupuncture, I do Bikram Yoga.’

For some, ongoing recovery involved incorporating the TC program’s morning routine into their lives. One participant attributed the core of their addiction problems to this lack of stability and routine.

‘I would just get up and do my morning program which was brisk walk, some exercise, come home and eat, basically. Which would set me up you know um for the day…’

#### Reduced substance use

Ten participants were abstinent at the time of the interview. Five had remained abstinent since leaving the program, ranging from one to four years. For many, the achievement they are most proud of was the accomplishment and maintenance of abstinence. Most attributed their abstinence in some way to the program, in addition to external sources such as social supports, ongoing DA treatments and employment.

‘… I haven’t touched a drink or drugs since leaving the <rehab organisation>.’

For some, abstinence was interrupted by lapses or relapse but overall these were much shorter than previous relapses. These shorter periods were seen as success because participants reported using skills learnt at the program to better recognise the early signs and halt the progression of the lapse before losing control.

‘I often use the knowledge that I gained while I was in the <rehab organisation>, like daily…’

#### Improved physical and psychological health

Ten participants reported an improvement in physical health, rating it as good or excellent. Two participants rated their physical health as fair or poor because of the desire to lose weight or increase fitness. Many reported improvements in psychological health and outlook on life. Some felt they had gained the ability to listen and be honest with themselves and others.

‘… I was just happy that my life looked like it was going to start going in a different direction.’

#### Relationship success

Most participants reported the positive influence of relationships after leaving the program. They began to form close relationships with new people and rebuild existing relationships. Some participants became a source of support for others.

‘…the ability to be able to listen and be honest with myself … and be able to share other people’s stories and listen to them to be a support.'

However, initial re-integration into the family model was quite challenging for one participant.

‘…you know just because I’m clean, not everyone’s jumping for joy and patting me on the back about that. There’s a lot of damage that’s done in active addiction. So dealing with some of the family members um distain, for lack of better words, of my behaviour while I was using that’s been hard.’

One participant turned to helping others as a way of ignoring their own trauma.

‘I tried to make my situation that I was helping other people so that I didn’t have to look at my own stuff.’

#### Employment success

Participants recognised the positive role that the program had played in developing the skills necessary to obtain and maintain employment or study after leaving the program.

‘…I wouldn’t have been able to get this job without having gone through the <TC program>.’

‘They may have lost me for 6 months but they’ve actually got a better employee.’

Eight participants reported volunteering after leaving the program, particularly in drug rehabilitation. Participants found helping and supporting others rewarding. Of the remaining five who were not volunteering, one was exploring options, two were gainfully employed and one was medically retired. For five participants, working in a volunteer position subsequently allowed them to attain paid employment.

‘…from volunteer work I ended up getting a job in drug and alcohol rehab, working with women and children in a residential environment.’

For some, employment has also helped to maintain abstinence.

‘… If I’m busy and being productive I’m not likely to smoke pot…’

## Discussion

The current study revealed a complex interplay of factors contributing to early TC withdrawal, and the perceptions of successful recovery from a lived experience perspective and how this was shaped by employment and volunteering. Eleven main themes were identified. Three relating to reasons for joining the program, which connected with ultimate withdrawal from the program: Pre-program existing relationships, pre-program employment situation and needing a ‘circuit breaker’ in their life. Three relating to reasons for early withdrawal: TC program characteristics, relationships during the program and planning future employment. Five relating to perceptions of successful recovery: Improved understanding of their addiction, reduced substance use, improved physical and psychological health, relationship success and employment success.

### Reasons for early withdrawal

#### To understand the reasons for early withdrawal, it was necessary to understand the reasons for entering the TC program.

Backgrounds of childhood trauma, abuse, isolation and an early age of first substance use were common amongst participants. Years of addiction culminated in the recognition of the need for an ‘extreme’ measure such as a long-term TC program, which was also influenced by pre-existing relationships and employment situation. The suffering associated with hitting rock bottom is a strong motivator for addiction recovery as individuals are forced to reassess their life and seek help [15]. The decision to leave treatment early is also heavily influenced by motivation to make positive changes in one’s life [10, 15]. Similarly, lower perceived likeliness of completing the program at intake was associated with lower completion rates, whilst having fewer stressful life events was a positive predictor of completion [9]. Indeed, our participants had left treatment early and most described significant life stressors.

The reasons for early withdrawal were varied. Many participants described aspects of the TC program as challenging, such as feeling confronted by the intensity of the program, disagreement with the program rules or struggling with the isolation. Others described a misunderstanding of the program or feeling ready to leave. Relationships and work commitments were also contributory factors. These findings are consistent with Chen et al.’s [11] who found program dissatisfaction and antagonistic staff and adolescent interactions core reasons for early exit. However, the current study found that relationships can have both positive and negative effects on program retention. The role of relationships are complex and their influence changes overtime. Prior to the TC program, relationships played an important role in both maintaining addiction and the decision to seek treatment. During the program, relationships with staff and fellow residents, as well as family outside the treatment environment, were particularly important drivers in the decision to stay or leave. Consistent with Landrum et al. [10], relationships can both positively and negatively affect treatment retention. Apart from Landrum et al. [10], other research has not shown this dichotomy, though this other research has focused on predictors or reasons for early withdrawal, not protective retention factors [11].

### Perceptions of successful recovery

Consistent with Aslan’s [12] conclusion that *“unsuccessful” can be successful*, the current study results show that individuals who leave the TC program early can still experience recovery success, regardless of whether they left voluntarily or were asked to leave involuntarily. Participants in the current study reported improvements in relationships, psychological and physical health, stability, employment and volunteering, mirroring findings that recovery success extends beyond abstinence [4, 5, 7, 12, 13].

In line with previous research [12, 13], abstinence played an important role in perceptions of successful recovery. Whilst some participants experienced lapses, many reported using the skills learnt from the TC program to better recognise early warning signs and break the cycle before losing control and falling back into active addiction. Lapses are part of unlearning chronically habitual behaviour and should be used as a learning experience [16].

Participants reported improved trigger recognition and understanding of recovery as a journey, as described by Best [17] “recovery is a process rather than an event and it is often characterised as a journey”. TC programs are structured to encourage reflective practice and this is shown in the current results, as the understanding of recovery as a journey was not fully appreciated until reflecting on the TC program and implementing these skills into their life outside the program. Laudet [13] also found that recovery is a process of growth, leading to improved financial and living conditions, physical and/or mental health and social networks.

All participants had engaged in further treatment, compared to Aslan’s [12] 85%. The strong theme of an improved understanding of recovery as a journey in the current study may account for these differences. The types of treatment were very similar, with the most commonly reported being support groups such as AA and NA used in conjunction with other treatment.

Positive relationships defined perceptions of successful recovery after leaving the program. Skills learnt from the program helped to rebuild emotional connections, which continue to positively influence their recovery. Results of the current study parallel what is shown through TC study outcomes reviews [4, 5]. However, it should be noted that some people continued to struggle after leaving the program and reported difficulties reconnecting with family and putting others above their own needs so they don’t have to deal with their own traumas.

### The link between employment, early withdrawal and recovery

The secondary aim was to explore how employment and volunteering related to early exit and perceptions of successful recovery. Employment, like relationships, showed varying influence across different time-points. Prior to the TC program, many reported difficulty attaining and maintaining jobs due to substance use. On the contrary, for some work was an enabler for substance use due to working in remote areas leading to social isolation, working in pubs and clubs or having the finances to afford buying substances. Commitments and motivation to find work because they felt ready to work again were reasons for leaving the program early. After leaving the program, participants reported improved self-worth and feeling able to contribute to society through study, volunteer work and paid employment, which was heavily represented in perceptions of successful recovery. These results are consistent with Vanderplasschen et al.’s [5] review of TC study outcomes and Aslan [12], who found TC residents were actively engaging in volunteering and paid employment after leaving the program.

### Strengths and limitations

A qualitative approach allowed for richer data on individual reasons and challenges. Telephone interviews were used for feasibility. Given the sensitive nature of the topic this approach may have afforded more honest data. One participant discussed feeling more comfortable speaking honestly when not face-to-face, which may reflect the view of many because of associated stigma. No studies have previously looked at the extended definition of what success looks like from ex-clients point of view in an Australian context.

Recollection bias and accurate recall may have been difficult for individuals who left the program four years ago compared to one year ago, however similar themes emerged regardless of time since leaving. Similarly, selection bias was an initial concern of the researchers, that only individuals who were abstinent and doing well would be willing to take part, however this was not the case. There was a reasonable spread of participants that dropped out of the program early and those that stayed. About 50% of participants stayed less than eight weeks. We had one participant that stayed less than two weeks, two that stayed between two and four weeks and four that stayed between five and eight weeks. Seven men and six women participated in the study. This was similar to the male-female ratio of those that have taken part in the residential programs at the residential rehabilitation service provider from which our participants were sampled. This may be different compared to other organisations and readers should take this into account when interpreting the results in the light of their own organisations client base. The validity of self-report data may be threatened by social desirability bias, though telephone interviews potentially countered this. Self-report among substance users has been investigated previously and found to have acceptable levels of validity and reliability [18–21]. Additionally, research has demonstrated that telephone interviews compared to face-to-face interviews can be used reliably among people with mental health disorders [22].

### Implications and recommendations

The current research illustrates the beneficial impact of the interpersonal skills training implemented in TC programs. It supports the program’s role in addressing recommendations of the National Drug Strategy [2], improvements in social competence training and increased community activity engagement. The study also shows TCs to be an effective DA treatment option for individuals who partially completed the program. This may be due to a shift in the way people view recovery, away from being defined solely by abstinence towards a more holistic view. This may include supporting residents to not only focus on abstinence but also to identify how they can actively contribute to the community, through studying, volunteering or finding employment. Although care should be taken, that this happens in a supportive and safe environment. Future research could utilise a mixed-method approach to clarify the relationships between predictors of and reasons for early withdrawal, outcome measures and perspectives of recovery success.

Many participants in the current study reported their substance use began at a young age. Future policy may look to target education around addiction at children. This education may draw parallels from other chronic conditions with a relapsing course or the anti-smoking campaigns; ‘never give up giving up’ [23].

## Conclusion

In conclusion, reasons for leaving treatment early are multi-faceted and revolve around positive and negative relationships, planning future employment, and program characteristics. The influence that each plays on their decision to leave early is varied and determined by the value they assign it. Perceived success extends far beyond achieving and maintaining abstinence to encompass improved relationships, psychological and physical wellbeing, understanding of addiction and employment, studying and volunteering. Self-worth and feeling able to contribute to society through employment, studying and volunteering were perceived to be essential elements of successful recovery. For many, it was only after leaving the TC program that they began to understand and appreciate the skills they had learnt and recognised the importance of ongoing DA treatment. Clinicians, policy makers and program developers should use the extended definition of successful recovery from the ex-clients perspective when determining the clinical and economic effectiveness of TC programs.

## Abbreviations

AA: Alcoholics Anonymous
DA: Drug and Alcohol
NA: Narcotics Anonymous
TC: Therapeutic communities
